# In Vitro Assessment of Selected Postbiotic Substances Against Methicillin-Resistant and Methicillin-Susceptible *Staphylococcus* spp. and *Mammaliicoccus* spp. of Bovine Mastitis Origin

**DOI:** 10.3390/ani16091422

**Published:** 2026-05-06

**Authors:** Mariola Bochniarz, Joanna Kowalik, Aneta Nowakiewicz, Aleksandra Trościańczyk, Agata Hahaj-Siembida, Katarzyna Michalak, Dorota Pietras-Ożga, Łukasz Adaszek, Andrea Lauková

**Affiliations:** 1 Sub-Department of Veterinary Microbiology, Department of Preclinical Veterinary Sciences, Faculty of Veterinary Medicine, University of Life Sciences, Akademicka 12, 20-033 Lublin, Poland; joanna.kowalik@up.edu.pl (J.K.); aneta.nowakiewicz@up.edu.pl (A.N.); aleksandra.troscianczyk@up.edu.pl (A.T.); agata.hahaj-siembida@up.edu.pl (A.H.-S.); 2 Department of Epizootiology and Clinic of Infectious Diseases, Faculty of Veterinary Medicine, University of Life Sciences, Gleboka 30, 20-612 Lublin, Poland; katarzyna.michalak@up.edu.pl (K.M.); dorota.ozga@up.edu.pl (D.P.-O.); lukasz.adaszek@up.edu.pl (Ł.A.); 3 Centre of Biosciences of the Slovak Academy of Sciences, Institute of Animal Physiology, Šoltésovej 4-6, 040 01 Košice, Slovakia; laukova@saske.sk

**Keywords:** cows, subclinical mastitis, postbiotic substances, nisin, methicillin-resistant *Staphylococcus* spp., *Mammaliicoccus* spp.

## Abstract

The growing problem of antibiotic resistance poses one of the greatest threats to modern medicine, making the search for new, alternative strategies to treat bacterial infections a matter of priority. The aim of the study was to evaluate the antimicrobial activity of selected postbiotic substances (PS) and nisin against methicillin-susceptible (MS) and methicillin-resistant (MR) strains of coagulase-negative staphylococci (NAS) and mammaliicocci. The study was conducted on 24 strains of coagulase-negative staphylococci and mammaliicocci isolated from milk collected from Holstein-Friesian (HF) cows with subclinical mastitis. In vitro, PS/EMo (100–400 AU/mL) and PS/Eas (100–200 AU/mL) were the most effective, inhibiting the growth of all MS and MR strains of NAS and *Mammaliicoccus* spp. (100.0%). A high percentage of strains were also sensitive to postbiotic substances produced by *Lactococcus lactis* subsp. *lactis* PS/MK2/8 (400–1600 AU/mL), PS/MK1/3 (100–400 AU/mL), and nisin (100–25,600 AU/mL), both in MR strains (94.1%, 82.4%, 88.2%, respectively) and in MS strains (100.0%, 85.7% and 100%, respectively). PS/Esach (100–25,600 AU/mL) inhibited the growth of all strains, including the *mec*A gene-positive mammaliicocci strains (100.0%), but was slightly less effective against the MS strains (64.7%). The lowest activity was observed with postbiotic substances produced by *Enterococcus faecium,* PS/4231 (100–1600 AU/mL) and PS/9296 (100–6400 AU/mL), which inhibited the growth of 3 out of 17 MR strains (17.65%). Among the *Mammaliicoccus* strains with the *mec*A gene, postbiotic substances derived from strains PS/4231 and PS/9296 inhibited the growth of 42.9% and 28.6% of the strains, respectively. Four tested strains were sensitive to all postbiotic substances (MSC9, MSC11, MSC12 and SCH3). This study confirms that postbiotic substances and nisin inhibit the growth of staphylococci isolated from the milk of cows with subclinical mastitis, including methicillin-resistant strains.

## Simple Summary

Mastitis is one of the most common diseases affecting dairy cattle and has a significant impact on the physico-chemical properties and composition of milk, as well as on animal welfare. The increasing antibiotic resistance of bacteria means that more and more strains are no longer responding to available drugs, which drastically limits therapeutic options. Especially, methicillin-resistant staphylococci, which are resistant to all β-lactam antibiotics, pose a particularly serious problem, often accompanied by resistance to other antibiotic groups (multidrug resistance). Therefore, it is necessary to develop new alternatives for treating infections that will reduce antibiotic use. One of the most promising areas of research is bacteriocins which are proteinaceous substances synthesized on ribosomes by both Gram-positive and Gram-negative bacteria and that have a bactericidal effect (eliminating microorganisms) or a bacteriostatic effect (inhibiting the growth of some microorganisms).

## 1. Introduction

Mastitis is one of the most common diseases affecting dairy cattle and has a significant impact on the physico-chemical properties and composition of milk, as well as on animal welfare [1,2,3]. It causes significant economic losses for farmers, including the costs of diagnostic tests, treatments, veterinary services, removal of sick animals from the herd, and decreased quality of the product sold [4]. Furthermore, it poses a potential risk to human health due to the possibility of zoonotic transmission and the rising incidence of antibiotic resistance [5]. Mastitis in cows can be caused by various pathogens, including bacteria, fungi, viruses, and algae [2,6,7,8]. However, it should be emphasized that the most common cause of mastitis is bacteria belonging to the genus *Staphylococcus.* They play a particularly important role, especially coagulase-positive *Staphylococcus aureus* [9]. Currently, a significantly higher percentage of mastitis cases are caused by coagulase-negative staphylococci, also known as non-aureus staphylococci (NAS), which are widespread in the natural environment and colonize the mucous membranes and skin of both animals and humans [10,11,12,13,14]. The most common NAS species isolated from the milk of cows with mastitis include *S. haemolyticus*, *S. chromogenes*, *S. epidermidis*, *S. warneri*, *S. cohnii*, *S. simulans*, *S. hominis*, *S. capitis*, and *S. xylosus* [15,16]. However, five NAS species that were previously classified in the genus *Staphylococcus* have been reclassified into the genus *Mammaliicoccus* based on 16S rRNA gene sequence analysis (*M. sciuri*, *M. fleurettii*, *M. lentus*, *M. stepanovicii,* and *M. vitulinus*) [17].

*Mammaliicoccus* spp. are Gram-positive, catalase-positive, non-spore-forming cocci characterized by aerobic or facultative anaerobic growth and variable oxidase activity. They are part of the normal microbiota of humans and animals and are considered potentially opportunistic pathogens capable of causing infections [18]. Similar to NAS, mammaliicocci can cause mastitis in various animal species and can also transfer resistance genes to other pathogenic microbiota, thereby contributing to the spread of multidrug resistance [19]. Especially, methicillin-resistant staphylococci, which are resistant to all β-lactam antibiotics, pose a particularly serious problem, often accompanied by resistance to other antibiotic groups (multidrug resistance). The mechanism of methicillin resistance involves the acquisition of the *mec*A gene, which encodes a modified enzyme protein (PBP2a), responsible for peptidoglycan biosynthesis in the bacterial cell wall [20,21]. For many years, the issue of MRSA was limited to hospitalized patients; strains isolated in hospitals are called HA-MRSA (hospital-acquired MRSA). However, in recent years, new groups of staphylococci have been isolated from the natural environment, known as CA (community-acquired) and FA/LA (food animal/livestock-associated) methicillin-resistant staphylococci [22]. The occurrence of these pathogens, which are also resistant to a number of other antibiotics, poses a potential risk to human and animal health [23]. To combat antibiotic resistance, it is essential to discover new, effective antibacterial agents of natural origin. Bacteriocins—antimicrobial peptides or proteins (AMPs) produced by certain bacteria with bactericidal or bacteriostatic effects represent a group of compounds with potential as both new-generation antibiotics and natural food preservatives [24].

Bacteriocins are proteinaceous substances synthesized on ribosomes and produced by both Gram-positive and Gram-negative bacteria. They exhibit antimicrobial activity and are usually secreted outside the bacterial cell [25]. The history of bacteriocins dates back to the early 1920s, with their antibacterial activity first discovered in 1928. In the 1960s, the first bacteriocin, nisin, was discovered, produced by the strain *Lactococcus lactis* subsp. *lactis* MCFB497 [26], and was later purified and recognized as a food preservative by the FAO/WHO in 1969 [27]. Bacteriocins either have a bactericidal or a bacteriostatic effect. These substances can have a broad inhibitory spectrum or a narrow one targeting specific species. As research on bacteriocins has advanced, a distinction has emerged between two categories of proteinaceous antimicrobial substances: postbiotic substances (PS), which have not been purified to homogeneity, and bacteriocins sensu stricto, defined as peptides purified to homogeneity [28,29,30,31,32]. According to ISAPP (International Scientific Association for Probiotics and Prebiotics), postbiotic substances are generally defined as preparations of inactive microorganisms and/or their components that provide health benefits to the host [28]. They include a broad range of metabolites, such as organic acids (lactic, acetic, and propionic), exopolysaccharides, enzymes, short-chain fatty acids, bioactive peptides, vitamins, and antimicrobial protein substances [30]. Substances referred to as bacteriocin-like inhibitory substances (BLIS), which have not been purified to a level of homogeneity but exhibit inhibitory activity against pathogens, are currently classified as postbiotic substances [33,34].

The aim of the study was to evaluate the antimicrobial activity of seven selected postbiotic substances and one lantibiotic bacteriocin-nisin against methicillin-susceptible (MS) and methicillin-resistant (MR) strains of coagulase-negative staphylococci and mammaliicocci isolated from the milk of cows with subclinical mastitis.

## 2. Materials and Methods

### 2.1. Classification of Animals for Study and Milk Sampling

Twenty-four strains of coagulase-negative *Staphylococcus* spp. and *Mammaliicoccus* spp. isolated from the milk of Holstein-Friesian (HF) cows with subclinical mastitis on one farm located in the Lublin Province (Poland). The herd consisted of 120 dairy cows. The cows were kept in a tie-stall system, fed a TMR (Total Mixed Ration), and straw was used as bedding material. The cows were milked twice a day using a high-line system, which is a type of milking pipeline system in which the milk pipeline is installed above the cows’ backs, usually above the milking stalls or along the ceiling of the barn. The average milk yield of the cows was 8610 kg per year. The average somatic cell count in the collected milk was 388,000 cells/mL of milk.

During morning milking, the California Mastitis Test (CMT) was performed as standard. If the test result is positive, a clinical examination of the cows was performed, and milk samples were collected for bacteriological testing and somatic cell count (SCC) determination. Before collecting milk samples, the teat skin was washed with water and then disinfected with 70% alcohol. The initial streams of milk were discarded. Milk for bacteriological testing was collected from the middle part into sterile, properly labeled tubes. The milk samples collected during morning milking were transported to the Department of Microbiology at the Faculty of Veterinary Medicine, University of Life Sciences in Lublin, and stored at 4 °C.

### 2.2. Classification of Mastitis Form

In cases of subclinical mastitis, there are no visible changes in milk or the udder, and no systemic symptoms are observed; however, the condition is diagnosed by an SCC > 200,000 cells/mL in milk and the presence of microorganisms in milk, as detected through microbiological testing. The SCC was measured using Somacount FC (Bentley, KS, USA).

### 2.3. Bacteriological Examination

The sensitivity assessment of seven selected postbiotic substances and one lantibiotic bacteriocin, nisin, was conducted on 24 strains of coagulase-negative *Staphylococcus* and *Mammaliicoccus* isolated from the milk of Holstein-Friesian (HF) cows with subclinical mastitis. Milk samples were thoroughly mixed, and then 10 µL of milk was inoculated onto agar medium with the addition of 5% defibrinated sheep blood (Columbia agar, Oxoid, Basingstoke, UK). The medium was incubated at 37 °C for 24 h under aerobic conditions. The initial identification involved assessing bacterial colony morphology, performing a catalase test, and preparing Gram-stained microscopic slides. Next, individual bacterial colonies grown on Columbia agar were inoculated onto differential media: Mannitol Salt Agar (Oxoid, Basingstoke, UK) and Baird-Parker (Oxoid, Basingstoke, Hampshire, UK). These media were used to differentiate staphylococci. Incubation was carried out under aerobic conditions at 37 °C for 24 h. Additionally, a test for the presence of the clumping factor (CF) was performed using the Staphytec Test (Oxoid, Basingstoke, UK), which differentiates between coagulase-positive and coagulase-negative staphylococcal strains. Pure cultures of isolated strains were identified using Matrix-assisted laser desorption/ionization time-of-flight mass spectrometry (MALDI-TOF MS) [35].

### 2.4. Evaluation of Methicillin Sensitivity in the Strains of Coagulase-Negative Staphylococci and Mammaliicocci

Phenotypic resistance to methicillin in coagulase-negative staphylococci and mammaliicocci was assessed using the disk diffusion method with a 30 µg cefoxitin disk. A bacterial suspension with a density of 0.5 on the McFarland scale was applied to Mueller-Hinton agar using a sterile swab. The results were interpreted after 24 h of incubation at 35 °C. Strains were considered resistant if an inhibition zone was ≤24 mm and sensitive if it was ≥25 mm, in accordance with CLSI (2009) recommendations [36].

### 2.5. Detection of the Presence of the mecA Gene Using Polymerase Chain Reaction (PCR)

The presence of the *mec*A gene was detected using the PCR method (Table 1). DNA from bacterial cells of NAS and *Mammaliicoccus* strains was extracted using the PCR Mix Plus Red (A&A Biotechnology, Gdańsk, Poland) according to the manufacturer’s instructions. The next step of the study was to verify the presence of the *mec*A gene in the genome of isolated strains using standard PCR. The primer sequences and PCR conditions were determined based on the available data [37]. Reactions were carried out in a thermocycler (TPersonal thermal cycler—Biometra GmbH, Goettingen, Germany).

### 2.6. Sensitivity to Postbiotic Substances of Selected Coagulase-Negative Staphylococci and Mammaliicocci

The antimicrobial activity was tested using an agar spot test as described by De Vuyst et al. [38]. An overnight culture of the indicator/tested strains (200 µL, with an absorbance at A_600_ between 0.6 and 1.0) was added to 4.0 mL of semi-solid Brain Heart agar (0.7%, *w*/*v*, Difco, Franklin Lakes, NJ, USA). This solid medium (BHA, 1.5% *w*/*v*, Difco) was overlaid with semi-solid agar containing the strain. The volume of 10 µL of each serial dilution of the postbiotic substance was spotted onto the surface of soft agar containing the bacterial/indicator strain, and the plate was incubated at 37 °C for 18 h. The postbiotic substances and nisin were diluted in phosphate buffer (pH 6.5, 1:1). After incubation, the zones of bacterial growth inhibition were evaluated. Inhibition activity was expressed in arbitrary units per milliliter (AU/mL) as the reciprocal of the highest twofold dilution of postbiotic substances and nisin, showing complete inhibition of the tested strain. All tests were repeated twice. The following postbiotic substances and lantibiotic bacteriocin nisin were used (Table 2): nisin, a lantibiotic bacteriocin prepared as previously described by Lauková et al. [39]. The postbiotic substances used were produced by the strains isolated, characterized, and studied at the Laboratory of Animal Microbiology, Center of Biosciences of the Slovak Academy of Sciences, Institute of Animal Physiology in Košice, Slovakia, and characterized at the same workplace location. The postbiotic substance PS/MK1/3 is produced by the strain *Lactococcus lactis* MK1/3, isolated from raw goat milk (accession number ON114093 in GenBank) as reported by Lauková et al. [40]. PS/MK2/8 (accession number PQ158272 in GenBank) is produced by *Lactococcus lactis* MK2/8, also isolated from raw goat milk [41]. PS/Ent 4231 is produced by the ruminal strain *Enterococcus faecium* CCM4231 [42]. Silage strain *E. faecium* EF9296 produces PS/Ent9296 [43]. PS/EMo/1-1Nik is produced by the strain *E. moraviensis* EMo1-1Nik from the buccal mucosa of Slovak warm-blood horses (accession number MW326085 in GenBank) [44]. *E. asini* Eas 1/11D27, originating from horses of the Slovak breed Norik from Muráň (accession number in GenBank MN822908), produces PS/EntEas [45]. PS/Esach is produced by *E. saccharolyticus* Es 3/11 D27 (accession number MN822909 in GenBank), isolated from the inner part of the auricle mucosa of a clinically healthy mare of Norik breed from Muráň [46].

## 3. Results

The bacteriological examination of milk sampled from cows with subclinical mastitis identified 13 strains of coagulase-negative staphylococci belonging to the genus *Staphylococcus*: *S. cohnii* (1 strain), *S. equorum* (1 strain), *S. haemolyticus* (1 strain), *S. saprophyticus* (2 strains), *S. simulans* (2 strains), *S. succinus* (3 strains), *S. xylosus* (3 strains), and 11 strains belonging to the genus *Mammaliicoccus*: *M. sciuri* (10 strains) and *M. vitulinus* (1 strain). Among the identified strains, 7 methicillin-resistant strains (MR) were detected: 6 of *M. sciuri* (MSC1, MSC7, MSC8, MSC9, MSC10, and MSC19) and one strain of *M. vitulinus* (MV19). The remaining 17 strains were methicillin-susceptible (MS): one strain of *S. cohnii* (SCH3), one strain of *S. equorum* (SQ21), one strain of *S. haemolyticus* (SHae4), two strains of *S. saprophyticus* (SP6 and SP24), four strains of *M. sciuri* (MSC2, MSC5, MSC11, and MSC12), two strains of *S. simulans* (SM27 and SM28), three strains of *S. succinus* (SS13, SS14, and SS25), and three strains of *S. xylosus* (SX17, SX20, and SX23).

### 3.1. Sensitivity to Postbiotic Substances of Methicillin-Susceptible NAS and Mammaliicoccus Strains

Postbiotic substance PS/EMo produced by *Enterococcus moraviensis* EMo 1-1Nik inhibited indicator strains with inhibitory activity in the range from 100 to 400 AU/mL. PS/Eas produced by *E. asini* Eas 1/11D27 was found to be the most effective (100–200 AU/mL, inhibiting the growth of all methicillin-susceptible NAS and *Mammaliicoccus* spp. (100.0% of strains, Table 3). A high percentage of strains were also sensitive to PS MK2/8, PS/MK1/3 (produced by *Lactococcus lactis* subsp. *lactis* MK2/8, MK1/3), and nisin (94.1%, 82.4%, 88.2%, respectively) with an inhibitory activity ranging from 400 to 1600 AU/mL and, 100–400 AU/mL and 100–25,600 AU/mL, respectively. The antimicrobial activity of PS/Esach, obtained from *E. saccharolyticus* Es 3/11D27, was comparatively weaker, affecting 11 of 17 indicator strains (64.7%) at concentrations between 100 and 25,600 AU/mL. The lowest activity was noted in the case of postbiotic substances PS/4231 and PS/9296 produced by *E. faecium* CCM4231 and EF9296. They inhibited the growth of only 3 of 17 strains (17.65%) with inhibitory activity of 100–1600 AU/mL and 100–6400 AU/mL, respectively (Table 3). Among the strains tested, *M. sciuri* MSC11, MSC12, and *S. cohnii* SCH3 were sensitive to all postbiotic substances (Table 3).

### 3.2. Sensitivity to Postbiotic Substances of Methicillin-Resistant Mammaliicoccus Strains

The highest efficacy against *mec*A gene-positive strains showed PS/EMo (100–400 AU/mL), PS/Eas (100–200 AU/mL), PS/MK2/8 (100–1600 AU/mL), and lantibiotic bacteriocin nisin (100–25,600 AU/mL) (Table 4). PS/MK1/3 (100–400 AU/mL) inhibited 85.7% of methicillin-resistant (MR) strains, whereas PS/Esach (100 AU/mL) was effective against 57.1% of them. The highest percentages of resistant strains were found in PS/4231 (57.1%, inhibitory activity 100–200 AU/mL) and PS/9296 (71.4%, 100–800 AU/mL). One *mec*A gene-positive strain, *M. sciuri* MSC9, was sensitive to all postbiotic substances and nisin (Table 4).

## 4. Discussion

In recent years, there has been a significant increase in the number of infections caused by strains of bacteria resistant to antibiotics used to treat humans and animals. The risk of infection from pathogenic microorganisms resistant to commonly used antibiotics is currently one of the most serious dangers [47,48,49]. The rapid spread of multidrug-resistant bacteria, including strains resistant to all available antibiotics, is mainly caused by misuse and overuse of antibiotics in both human and animal populations [50,51]. It should be emphasized that the unjustified use of drugs on farm animals plays a significant role in this process, and the most frequently reported reason for the use of antibiotic therapy in cow breeding is mastitis [1]. The problem is continuously escalating and poses a serious threat to human health, as approximately 62.0% of isolated mastitis-causing agents are resistant to at least one antibiotic [52]. Therefore, it is necessary to develop new alternatives for treating infections that will reduce antibiotic use. Increasing emphasis is being placed on natural antimicrobial therapies. It has been documented that the use of probiotics (live, non-pathogenic microorganisms) and postbiotics (non-living bacteria, cell components, metabolites, or fermentation by-products) decreases the incidence of pathogens in large-scale animal breeding [53,54]. Research results indicate that postbiotic substances exhibit high antimicrobial activity, including the possibility of eradicating bacterial biofilm without negatively affecting obligate microbiota, are resistant to temperature and pH changes, and are characterized by low toxicity [55,56].

The most studied are lantibiotics produced, e.g., by certain strains of *Lactococcus lactis* subsp. *lactis* [57] such as nisin, which is active against many Gram-positive bacteria and is currently registered in the European Union (EU) under Regulation (EC) 1333/2008 as a food biopreservative (E 234) [58]. High activity of nisin has also been documented in treating various infections caused by multidrug-resistant strains of microorganisms, immunomodulation, and even anti-cancer therapies [59]. In our study, nisin effectively inhibited the growth of all *mec*A gene-positive strains and a large percentage (88.2%) of methicillin-sensitive strains of NAS and *Mammaliicoccus* spp. The results of our research align with those of other authors. Wu et al. [60] demonstrated that intramammary application of nisin at a dose of 2,500,000 IU for 3 days in cows with subclinical mastitis had bacteriological cure rates of 90.1% for *Streptococcus agalactiae*, 50.0% for *Staphylococcus aureus*, and 58.8% for coagulase-negative staphylococci. Similar results were obtained by Cao et al. [61], who studied the in vivo effects of nisin and compared it with that of gentamicin (GM). Their experiment showed that nisin offered a clinical cure rate similar to GM (90.2 vs. 91.1%). The high efficacy of nisin against bacteria isolated from cows with mastitis was also confirmed in studies by Kaczorek et al. [62]. These authors found that even a low concentration of nisin solution (≥9.76 IU/mL) can inhibit the growth of *Str. uberis*, *Str. agalactiae* and *Str. dysgalactiae*. Many studies are currently underway to enhance the activity of nisin. Field et al. [63] demonstrated in their research on bioengineered nisin derivatives that nisin A variants have improved antimicrobial activity against pathogenic staphylococci, including strains isolated from the milk of cows with mastitis, without negatively affecting the commensal microbiota of milk.

In addition to nisin, *Lactococcus lactis* (*L. lactis*) can also produce other postbiotic substances with antimicrobial properties. From the perspective of practical applications of postbiotic substances in the dairy industry, research on *L. lactis* MK1/3 (CCM 9209) and MK2/8 strains, isolated from raw goat’s milk by Lauková et al. [40,41], is particularly interesting. These strains have a favorable profile—they are susceptible to antibiotics, do not produce toxic enzymes (including β-glucuronidase), have a low ability to form biofilms, and tolerate bile and low pH. The postbiotic substances secreted by strains MK1/3 and MK2/8 primarily inhibit Gram-positive bacteria [40,41]. In our study, a high percentage of strains, both MR strains (94.1% and 82.4%, respectively) and MS strains (100.0% and 85.7%, respectively), were sensitive to PS/MK2/8 and PS/MK1/3.

The ability to produce postbiotic substances is not limited solely to lactic acid bacteria. Among postbiotic substances with documented antibacterial activity, Enterocin CCM 4231 (Ent 4231), produced by the ruminal strain *Enterococcus faecium* CCM 4231 plays a special role [42]. It was one of the first enterocins reported by Lauková and Czikková [64], which exhibits a broad spectrum of activity against Gram-positive bacteria, including *Listeria monocytogenes* (bactericidal effect) and *S. aureus* (bacteriostatic effect). The enterocin-producing strain 4231 was then used in numerous rabbit experiments in combination with sage extract, showing a beneficial effect on the intestinal microbiota and health parameters [65]. Another important postbiotic substance with inhibitory activity against Gram-positive bacteria, including *Enterococcus* spp. and *L. monocytogenes*, is produced by the *E. faecium* EF 9296 strain isolated from silage [43]. This strain was sensitive to ampicillin, erythromycin, tetracycline, rifampicin, and vancomycin, and only resistant to kanamycin. In this study, the postbiotic substances produced by *E. faecium* showed the weakest activity among those tested. PS/4231 and PS/9296 inhibited the growth of only 17.65% MR strains. Among the group of *mec*A gene-positive *Mammaliicoccus*, PS/4231 and PS/9296 were effective against 42.9% and 28.6% strains, respectively.

In the search for new sources of postbiotic substances, the species *Enterococcus moraviensis,* first isolated from surface waters, is especially noteworthy. Lauková et al. [44] described *E. moraviensis* EMo 1-1Nik strain isolated from the oral mucosa of Slovak warm-blood horses (99.93% similarity to NR113937.1). This strain is hemolysis-negative, lacks virulence factor genes, and has low biofilm formation ability. Up to 60.0% of EMo 1-1Nik colonies produced bacteriocins against *E. avium* EA5, reaching an activity of 800 AU/mL. Additionally, this substance is heat-stable and resistant to enzymatic processing. In our own study, PS/Emo inhibited the growth of all MS and MR strains of staphylococci and *Mammalicoccus* spp. (100.0% of strains).

An even less-known species with postbiotic potential is *Enterococcus asini,* first described by de Vaux et al. [66]. Lauková et al. [45] conducted pioneering research on the *E. asini* EAs 1/11D27 strain, isolated from the mucous membrane of the Slovak horse breed Norik from Muráň (99.86% similarity to NR113929.1, GenBank AN: MN822908). The EAs 1/11D27 strain is hemolysis-negative (ɤ-hemolysis), DNase-negative, gelatinase-negative, absent of virulence factor genes, sensitive to antibiotics, and does not produce toxic enzymes. The postbiotic substance of this strain inhibited the growth of 97.0% of tested indicator strains, with the highest inhibitory potential observed against staphylococci, enterococci, lactococci, and streptococci. It should also be emphasized that the growth of 9 of 10 Gram-negative bacterial strains was also inhibited, opening new perspectives for the use of PS in veterinary medicine. In our study, PS/Eas inhibited the growth of all MS and MR strains of staphylococci and *Mammalicoccus* spp. (100.0% strains).

Research on the microbiota of the Slovak horse breed Norik from Muráň horse has also identified the *Enterococcus saccharolyticus* Es 3/11 D27 strain, which is a valuable addition to our knowledge of the species diversity of enterococci colonizing the mucous membranes of equines [46]. The species *E. saccharolyticus*, originally described by Farrow et al. [67] as *Streptococcus saccharolyticus* and reclassified to the genus *Enterococcus,* is relatively rarely isolated from clinical material and has not yet been studied for its postbiotic potential. The detection of this species in the equine microbiome suggests that equids may harbor previously unknown strains of enterococci with potential bacteriocinogenic activity. In our study, more than half of the MR and MS strains (57.1% and 64.7%, respectively) were sensitive to PS/Esach.

## 5. Conclusions

In summary, our research confirms that postbiotic substances and nisin inhibit the growth of staphylococci isolated from the milk of cows with subclinical mastitis, including methicillin-resistant strains. Particularly promising are findings related to the postbiotic potential of lesser-known species such as *E. moraviensis* (EMo 1-1Nik), *E. asini* (EAs 1/11D27), and *E. saccharolyticus* (Es 3/11 D27), as well as the *L. lactis* MK1/3 and MK2/8 strains. Given the increasing problem of antibiotic resistance, bacteriocins and postbiotics appear to be a safe, effective, and environmentally friendly alternative for preventing and treating mastitis in cows, and warrant further intensive clinical research.

## Figures and Tables

**Table 1:** Sequences of the primer used in the study.

Gene	Primer Sequence (5′ Do 3′)	Product Size (bp)	Source
*mec*A	F: TCCAGATTACAACTTCACCAGG	162	Larsen et al. [37]
	R: CCACTTCATATCTTGTAACG		

**Table 2:** The postbiotic substances used in the study.

Substance	Producer Strain	Source	References
Nisin	*Lactococcus lactis* subsp. *lactis* MCFB497	Bozaris and Adams, 1999 [26]	Lauková et al. [39]
PSMK1/3	*L. lactis* MK1/3 AN ON114093	Raw goat milk	Lauková et al. [40]
PSMK2/8	*L. lactis* MK2/8 AN PQ158272	Raw goat milk	Lauková et al. [41]
PS/Ent4231	*E. faecium* CCM4231	Ruminal strain	Lauková et al. [42]
PS/Ent 9296	*E. faecium* EF9296	Silage strain	Marciňáková et al. [43]
PS/EMo	*E. moraviensis* EMo 1-1Nik MW326085	Buccal mucosa of Slovak warm-blood horses	Lauková et al. [44]
PS/Eas	*E. asini* Eas 1/11D27 MN822908	Slovak horse breed Norik from Muráň	Lauková et al. [45]
PS/Esach	*E. saccharolyticus* Es 3/11 D27 MN822909	Auricle mucosa of a clinically healthy mare of Norik breed from Muráň	Lauková et al. [46]

**Table 3:** Sensitivity to postbiotic substances of methicillin-susceptible NAS and *Mammaliicoccus* strains expressed in arbitrary units per milliliter (AU/mL).

	Nis	PS/MK1/3	PS/MK2/8	PS/4231	PS/9296	PS/EMo	PS/Eas	PS/Esach
MSC2	6400	400	1600	0	0	100	200	0
MSC5	6400	200	1600	0	0	400	200	100
MSC11	800	200	1600	1600	6400	400	200	200
MSC12	400	400	1600	100	800	100	100	100
SHae4	25,600	200	400	0	0	100	200	0
SX17	1600	100	1600	0	0	100	100	0
SX20	100	200	1600	0	0	100	100	0
SX23	3200	100	1600	0	0	100	200	100
SCH3	1600	400	1600	100	100	200	200	0
SP6	100	100	100	0	0	200	100	0
SP24	1600	100	1600	0	0	100	100	100
SQ21	1600	100	1600	0	0	100	100	100
SM27	0	0	100	0	0	100	100	25,600
SM28	0	0	0	0	0	100	100	12,800
SS13	800	400	1600	0	0	100	100	100
SS14	1600	0	800	0	0	200	100	100
SS25	3200	200	1600	0	0	100	200	100
Total	15/17 (88.2%)	14/17 (82.4%)	16/17 (94.1%)	3/17 (17.65%)	3/17 (17.65%)	17/17 (100.0%)	17/17 (100.0%)	11/17 (64.7%)

MSC—*Mammaliicoccus sciuri*; SHae—*Staphylococcus haemolyticus*; SX—*Staphylococcus xylosus*; SCH—*Staphylococcus cohnii*; SP—*Staphylococcus saprophyticus*; SQ—*Staphylococcus equorum*; SM—*Staphylococcus simulans*; SS—*Staphylococcus succinus*; PS—postbiotic substances.

**Table 4:** Sensitivity to postbiotic substances of methicillin-resistant *Mammaliicoccus* strains.

	Nis	PS/MK1/3	PS/MK2/8	PS/4231	PS/9296	PS/EMo	PS/Eas	PS/Esach
MSC1	12,800	400	1600	0	0	200	200	0
MSC7	800	100	400	100	0	200	100	0
MSC8	25,600	0	100	0	100	100	100	100
MSC9	3200	400	1600	200	800	400	100	100
MSC10	1600	200	100	100	0	200	100	100
MSC19	800	400	1600	0	0	400	100	100
MV22	100	100	1600	0	0	200	100	0
Total	7/7 (100.0%)	6/7 (85.7%)	7/7 (100.0%)	3/7 (42.9%)	2/7 (28.6%)	7/7 (100.0%)	7/7 (100.0%)	4/7 (57.1%)

MSC—*Mammaliicoccus sciuri*; MV—*Mammaliicoccus vitulinus*; PS—postbiotic substances.

## Data Availability

The original contributions presented in this study are included in the article. Further inquiries can be directed to the corresponding author.
